# Effect of Autologous Serum Eyedrops on Ocular Surface Disease Caused by Preserved Glaucoma Eyedrops

**DOI:** 10.3390/jcm9123904

**Published:** 2020-12-01

**Authors:** Ha-Rim So, Hae Young Lopilly Park, So-Hyang Chung, Hyun-Seung Kim, Yong-Soo Byun

**Affiliations:** Department of Ophthalmology, Seoul St. Mary’s Hospital, College of Medicine, The Catholic University of Korea, 222, Banpo-daero, Seocho-gu, Seoul 06591, Korea; dreamss2@hanmail.net (H.-R.S.); lopilly@catholic.ac.kr (H.Y.L.P.); chungsh@catholic.ac.kr (S.-H.C.); sara514@catholic.ac.kr (H.-S.K.)

**Keywords:** autologous serum eyedrops, dry eye disease, glaucoma, ocular surface disorder, toxicity

## Abstract

Autologous serum eyedrops (ASE) are effective in treating various ocular surface diseases, including damages induced by long-term use of preserved glaucoma eyedrops. However, there has been no study on whether ASE is effective without stopping the causative eyedrops. This retrospective observational study included 55 patients with ocular-surface diseases caused by long-term use of preserved glaucoma eyedrops: 18 patients who used ASEs for 2 months without discontinuing the use of glaucoma eyedrops (Group 1), 22 patients who used ASEs for 2 months, discontinuing the use of glaucoma eyedrops for the first month (Group 2) and 15 patients who used non-preservative artificial tears for 2 months, discontinuing the use of glaucoma eyedrops for the first month (Group 3). There were no intergroup differences in the baseline values of the Schirmer I test results, tear breakup time (TBUT), ocular surface staining (OSS) score, loss of the meibomian gland, meibum quality and ocular-surface disease index (OSDI). Group 1 showed significant differences in TBUT, OSS score and OSDI at 2 months when compared to the baseline values before treatment, while Group 2 showed significant differences in those values at both 1 and 2 months. There were no differences in any of the parameters at baseline, 1 month or 2 months in Group 3. Our result suggested that ASE is effective for treating ocular surface diseases caused by glaucoma eyedrops containing preservatives and its effects can be expected without interruption of glaucoma eyedrop treatment.

## 1. Introduction

The ocular surface, comprised of cornea, conjunctiva and eyelid, is the outmost layer of the eyes that is exposed to the external environment. Therefore, the ocular surface homeostasis is of critical importance to protect the inner structures of eyeball from trauma or pathogens and preserve the normal vision. Blinking, tear film, microbial commensal, ocular immunity and epithelial barrier are necessary to maintain the homeostasis and health of ocular surface. However, these defense mechanisms may be compromised in various diseases, leading to the severe discomfort and even visual impairment [1].

Glaucoma eyedrops are currently the first-line treatment for lowering intraocular pressure (IOP) and preventing disease progression. Despite the benefits of lower IOP for glaucoma management, it is burdensome for patients to maintain, indefinitely, the daily administration of eyedrops [2]. Additionally, chronic exposure to the active ingredients and preservatives of these drops, such as benzalkonium chloride (BAK), causes an adverse effect on the ocular surface [3,4]. Dry eye disease, superficial punctate keratitis, lid margin abnormality and meibomian gland dysfunction are more common among glaucoma patients using glaucoma eyedrops than among the general population [5].

There have been many efforts to reduce ocular complications in patients requiring long-term use of glaucoma eyedrops. Use of a sodium hyaluronate and taurine solution leads to significant improvement in a patient’s ocular surface condition with long-term use of preserved eyedrops [6,7]. Several glaucoma eyedrops have recently been made available in disposable, single-use packaging and use of preservative-free formulas significantly decreases the ocular-surface symptoms [8,9]. As an alternative to topical medications, surgical treatments such as laser trabeculoplasty, filtering surgery or trabeculectomy may be considered for patients experiencing severe drug toxicity [10]. However, these additional options are not yet available to all patients experiencing ocular surface complications.

Autologous serum eyedrops (ASEs) have been used to treat a variety of ocular surface diseases, including dry eye disease, Sjogren syndrome, persistent epithelial defects, neurotrophic keratopathy, exposure keratitis, recurrent corneal erosion, Stevens-Johnson syndrome and graft-versus-host disease, among others [11]. There is limited research on the effects of ASEs on ocular-surface diseases associated with glaucoma medications [12] and it remains unknown whether ASEs improve the ocular surface with continued use of glaucoma eyedrops. This is a practical issue for many patients that requires further study. Therefore, this study investigated whether ASEs are effective in treating ocular surface diseases caused by glaucoma eyedrops containing preservatives with continued use of the causative eyedrops.

## 2. Experimental Section

### 2.1. Patient Groups

The study protocol was approved by the Institutional Review Board of Seoul St. Mary’s Hospital (KC18RESI0813) and all procedures were conducted in compliance with the ethical standards of the institutional research committee and the Declaration of Helsinki. This retrospective, non-randomized, comparative trial included 55 patients treated for ocular surface diseases associated with the use of preserved glaucoma eyedrops.

The inclusion criteria were as follows—patients who felt chronic ocular discomfort that occurred after starting glaucoma eyedrops, who did not receive any treatment other than preservative-free artificial tears and who had corneal or conjunctival staining. The exclusion criteria were as follows: patients with a history of ocular surgeries within 6 months, acute inflammation with suspected infection and unknown origin or other comorbid anterior segment anomalies. If both eyes of a patient met the inclusion criteria, only the eye with the higher ocular surface staining (OSS) score was included; if both eyes had the same score, only the right eye was included.

**Figure 1:**
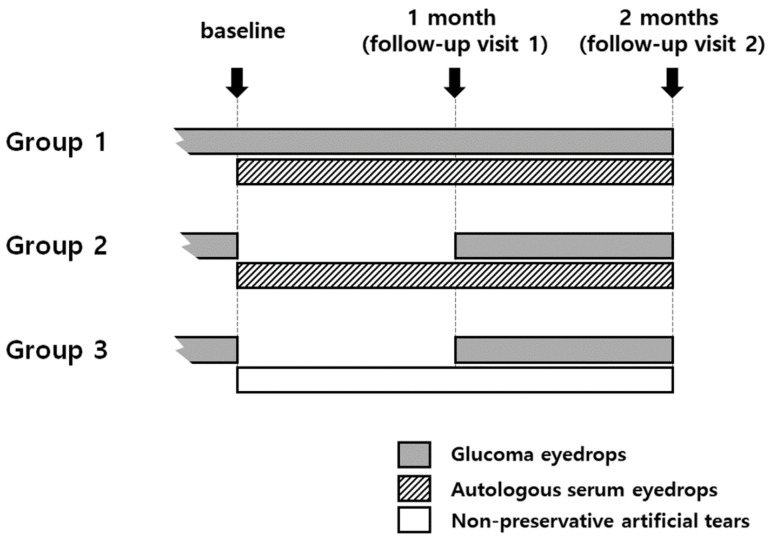
**Group definitions.** Group 1, autologous serum eyedrops (ASEs) for 2 months without discontinuing glaucoma eyedrops (*n* = 18); Group 2, ASEs for 2 months and discontinuing glaucoma eyedrops for the first month (*n* = 22); Group 3, preservative-free artificial tears for 2 months and discontinuing glaucoma eyedrops for the first month (*n* = 15).

The 55 patients were divided into three groups according to their use of ASEs and temporary discontinuation of glaucoma eyedrops containing preservatives: Group 1 (*n* = 18) used ASEs for 2 months without discontinuing the glaucoma eyedrop treatment; Group 2 (*n* = 22) used ASEs for 2 months and discontinued the glaucoma eyedrop treatment for the first month; and Group 3 (*n* = 15) used preservative-free artificial tears (0.1% Tearin free, DHP, Seoul, Korea) for 2 months and discontinued the glaucoma eyedrop treatment for the first month (Figure 1). Treatment of ocular surface disease was conducted in consultation with a glaucoma specialist (PHY) and with informed consent from the patient for treatment. Temporary discontinuation of glaucoma eyedrops was not implemented for patients with advanced glaucoma or unstable IOP or for those who did not wish it. ASEs were made from the patient’s peripheral venous blood under sterile conditions [13]. Whole blood collected in a tube without anticoagulants was centrifuged at 3500 rpm for 15 min to obtain the serum. The isolated serum was then diluted to 20% with preservative-free artificial tears (0.1% Hyaluronic acid, Tearin free, DHP, Seoul, Korea) and placed in a refrigerator for long-term storage. The ASEs were applied six times a day.

### 2.2. Objective Measures

All assessments were performed on the first visit before treatment (baseline) and at follow-up visits 1 and 2 months after treatment. The objective measures included the Schirmer I test results, tear breakup time (TBUT), OSS score, loss of the meibomian gland (meiboscore), meibum quality and ocular surface disease index (OSDI). Additionally, IOP was investigated to evaluate fluctuations caused by the 1-month discontinuation of glaucoma eyedrop treatment in Groups 2 and 3.

The application of a dye or irritant on the ocular surface for testing may affect the tear film and prevent accurate testing. For this reason, the Schirmer I test was performed first, then TBUT was evaluated using a fluorescein dye, followed by corneal and conjunctival staining patterns and the meibomian gland was evaluated last. For the Schirmer I test, Schirmer paper (Color BarTM, Eaglevision Inc., Memphis, TN, USA) was inserted into the outer 1/3 of the lower eyelids on both sides and maintained for 5 min with both eyes closed. Using a slit lamp microscope, fluorescein paper (Haag-Streit, Köniz, Switzerland) was applied to the conjunctiva and then the patient blinked for the TBUT measurement. The OSS score was measured according to the Sjogren’s International Collaborative Clinical Alliance registry ocular examination protocol [14]. The cornea and conjunctiva were examined with a slit lamp using fluorescein paper and then with lissamine green strips (Green Glo, HUB Pharmaceuticals, Rancho Cucamonga, CA, USA). Fluorescein-stained punctate epithelial erosions (PEEs) were counted and scored. Conjunctival dots stained with lissamine green in the interpalpebral bulbar conjunctiva were also counted and scored. The corneal score ranged from 0 to 6: (0) no PEEs; (1) 1–5 PEEs; (2) 6–30 PEEs; (3) >30 PEEs. Additional points were added as follows: (1) PEEs were found in the central portion of the cornea; (2) 1 or more filaments was seen anywhere on the cornea; or (3) 1 or more patches of confluent staining were seen on the cornea. The conjunctival score ranged from 0 to 3 (separately in the temporal and nasal bulbar conjunctiva): grade 0, 0–9 dots of lissamine green staining; grade 1, 10–32 dots; grade 2, 33–100 dots; and grade 3, >100 dots. The total OSS score ranged from 0 to 12. The grade of Meibomian-gland loss was measured from the upper lid using a slit lamp equipped with a non-contact infrared meibography system (0 = no loss; 1 = loss < 33%; 2 = loss 33–66%; 3 = loss > 66%) [15]. The quality of expressed secretion in Meibomian-gland dysfunction was evaluated as follows: 0, clear; 1, cloudy; 2, granular; 3, toothpaste [16]. All parameters measured at follow-up visits were compared to the baseline values in each group.

### 2.3. Statistical Analysis

ASEs were made from the patient’s peripheral venous blood under sterile conditions [13]. Whole blood collected in a tube without anticoagulants was centrifuged at 3500 rpm for 15 min to obtain the serum. The isolated serum was then diluted to 20% with preservative-free artificial tears (0.1% Hyaluronic acid, Tearin free, DHP, Seoul, Korea) and placed in a refrigerator for long-term storage. The ASEs were applied six times a day.

The data used to support the findings of this study are available from the corresponding author upon request.

## 3. Results

Patient demographics are presented in Table 1. There were no differences in age or sex predominance. Among all groups, normal-tension glaucoma was the most common diagnosis and the use of one type of glaucoma eyedrops was more common than the use of more than one type to lower the IOP.

**Figure 2:**
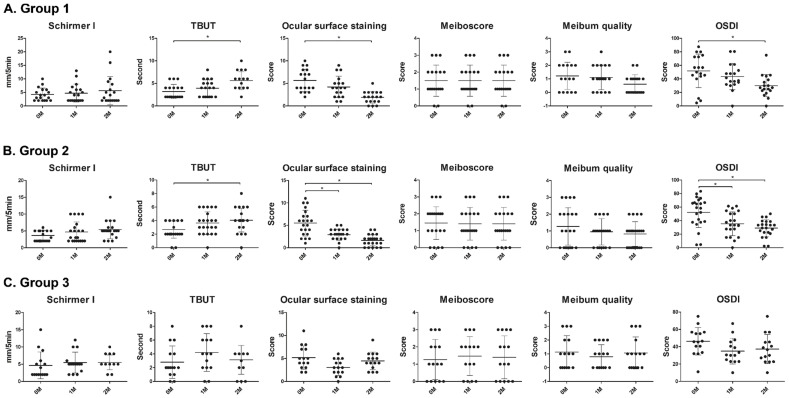
**Changes in ocular surface-related parameters during a 2-month observation period in each group.** Tear breakup time (TBUT), ocular surface staining (OSS) score and ocular-surface disease index (OSDI) were significantly improved in Groups 1 and 2 at 2 months when compared to the baseline and Group 2 also showed significant improvement in TBUT, OSS score and OSDI at 1 month. However, in Group 3, no changes were noted at 2 months in the Schirmer I test, meiboscore or meibum quality. The lines in the graph present the mean and standard deviation. * *p* < 0.05 by Dunn’s post-hoc comparison after Kruskal-Wallis test; Group 1, autologous serum eyedrops (ASEs) for 2 months without discontinuing glaucoma eyedrops (*n* = 18); Group 2, ASEs for 2 months and discontinuing glaucoma eyedrops for the first month (*n* = 22); Group 3: non-preservative artificial tears for 2 months and discontinuing glaucoma eyedrops for the first month (*n* = 15).

In Groups 1 and 2, more patients had used preserved glaucoma eyedrops for more than 1 year than for less than 1 year (83.3% and 77.3%, respectively). In Group 3, fewer patients had used preserved glaucoma eyedrops for more than 1 year (40.0%). At baseline, there were no intergroup differences in ocular-surface parameters, including the Schirmer I test (*p* = 0.8514), tear breakup time (TBUT) (*p* = 0.5057), ocular surface staining (OSS) score (*p* = 0.8423), meiboscore (*p* = 0.7581), meibum quality (*p* = 0.9009) and ocular surface disease index (OSDI) (*p* = 0.3412). Intragroup analysis showed no differences from baseline in Groups 1 and 3 at 1 month; Group 2 showed a significant increase in TBUT and decreases in OSS score and OSDI. At 2 months, Groups 1 and 2 showed a significant increase in TBUT and decreases in OSS score and OSDI; Group 3 did not show any significant changes in variables compared to baseline (Table 2, Figure 2). Changes in Schirmer I test, meiboscore and meibum quality were not found in any of the groups during the study period.

**Figure 3:**
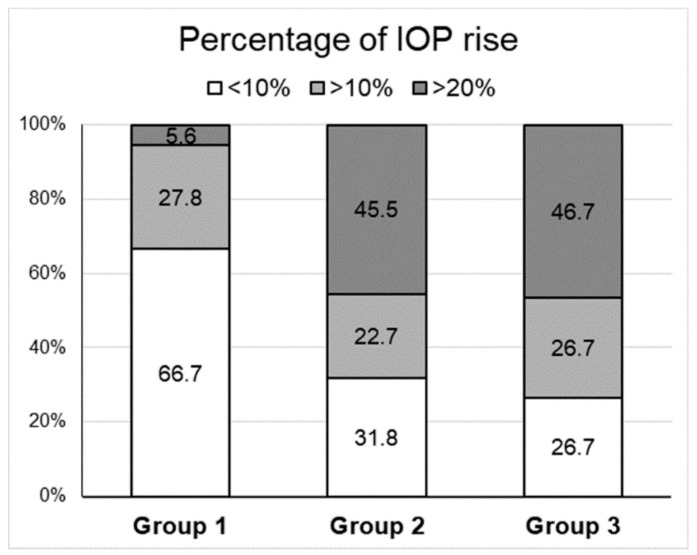
**Percentage of patients with increased intraocular pressure (IOP) after treatment in each group.** Group 1, autologous serum eyedrops (ASEs) for 2 months without discontinuing glaucoma eyedrops (*n* = 18); Group 2, ASEs for 2 months and discontinuing glaucoma eyedrops for the first month (*n* = 22); Group 3, non-preservative artificial tears for 2 months and discontinuing glaucoma eyedrops for the first month (*n* = 15).

After the 1-month discontinuation of glaucoma treatment, the IOP of patients in Group 2 had increased by more than 10% and >20% in 68.2% (15/22) and 45.5% (10/22) of the patients, respectively. The IOP of patients in Group 3 increased by more than 10% and 20% in 73.4% (11/15) and 46.7% (7/15), respectively, which is higher than that observed in Group 1 (in which patients continued the glaucoma treatment) (Figure 3).

## 4. Discussion

Our results showed that ASEs were effective in improving symptoms of ocular surface disease caused by preserved glaucoma eyedrops in patients who used ASEs for 2 months without discontinuing their treatment of glaucoma eyedrop containing preservatives (Group 1) and in patients who used ASEs while discontinuing their treatment of glaucoma eyedrops with preservatives for 1 month (Group 2). No improvements were noted in patients using preservative-free artificial tears while discontinuing their preserved glaucoma eyedrop treatment for 1 month (Group 3). In particular, the use of ASEs without temporary discontinuation of the glaucoma eyedrops containing preservatives led to significant improvements in TBUT, OSS score and OSDI at 2 months, although it took more time than required when using ASEs while temporarily discontinuing the glaucoma eyedrop treatment, which resulted in improvements in OSS score and OSDI at 1 month. This suggests that ASEs will help patients with advanced glaucoma who cannot discontinue use of their glaucoma medication. This finding is meaningful in that ASEs do not affect the treatment plan for glaucoma.

Many glaucoma eyedrops are used as the primary treatment for glaucoma; topical medication is used for long-term regulation of a patient’s IOP. This long-term medication use and the resulting exposure to preservatives inevitably leads to ocular surface diseases [17,18]. Most glaucoma eyedrops contain preservatives such as BAK, polyquaternium-1, stabilized oxychloro complex and sodium perborate. In particular, BAK can affect many ocular structures such as the corneal epithelium, conjunctiva and trabecular meshwork. It can also affect the lipid layer of the tear film [19]. In addition, it is reported that long-term use of prostaglandin analogs may alter conjunctival microbial flora [20]. The alteration of the normal flora can cause inflammation, delayed healing and susceptibility to pathogen invasion, followed by worsening of ocular surface diseases [1]. Approximately 49–59% of patients with glaucoma experience ocular surface diseases related to the use of glaucoma eyedrops but these problems have been under-recognized and under-treated to allow for better control of IOP. Ocular surface diseases have a significant impact on the quality of life of patients with glaucoma; they can also affect medication compliance and the success of the glaucoma therapy [21,22]. Many efforts have been made to reduce ocular surface complications caused by glaucoma eyedrops [23]: Preservative-free eyedrops, glaucoma eyedrops with less BAK, eyedrops containing alternative preservatives and the frequent use of artificial tears have helped to relieve patients’ symptoms.

Human serum contains many components such as vitamin A, epidermal growth factor, fibronectin and transforming growth factor-β, all of which are important for the proliferation, differentiation and maturation of the ocular surface epithelium [13,24,25]. According to a recently published study, ASEs have an effect on toxic epitheliopathy caused by the BAK in glaucoma eyedrops. Recently, Oh et al. reported the effects of ASEs on ocular surface diseases induced by glaucoma medication. They reported significantly lower OSDI, increased TBUT, recovered corneal sensation and reduced levels of matrix metallopeptidase 9 [12,26].

In the present study, we compared the continued use of glaucoma eyedrops with discontinuation of glaucoma eyedrops while using ASEs and found that ASEs were effective in treating toxic keratopathy even when glaucoma eyedrop treatment was maintained. Several studies [12,27] have suggested that BAK increases the levels of cytokines such as IL-6, IL-8 and IL-1β in the tears of patients using glaucoma eyedrops and that these inflammatory regulators cause ocular discomfort. Oh et al. have shown that the levels of these cytokines can further increase when corneal epithelial cells are destroyed and that they can induce inflammation by producing molecules called damage-associated molecular patterns (DAMPs). In the case of ASEs, this study hypothesized that the integrity of the corneal epithelial cells would increase, thereby reducing the production of inflammatory cytokines. This study also hypothesized that treatment with ASEs would be effective, even with continued use of glaucoma eyedrops. Our findings confirmed both of these hypotheses.

It has been reported that IOP-lowering eyedrops can lead to eyelid changes associated with meibomian-gland dysfunction [28]. Our results showed that the use of ASEs, with or without discontinuing glaucoma eyedrop treatment, had no effect on the morphology of the meibomian gland or the quality of the meibum, though it did result in an increase in TBUT. This may be due to irreversible changes in the meibomian glands over time [29].

The present study had several limitations. Due to small sample size and retrospective observational study design, glaucoma eyedrops related factor such as the type and frequency could not be analyzed in this study. Future study should exclude the confounding factors related to glaucoma eyedrops and glaucoma type and validate the effect of ASE through prospective study. Our findings have confirmed the little-known effect of ASEs on ocular surface diseases related to long-term use of glaucoma eyedrops containing preservatives. Furthermore, this study has demonstrated that a significant effect can be expected by adding ASEs without interrupting glaucoma eyedrop treatment.

## 5. Conclusions

ASE is effective in treating ocular-surface diseases caused by long-term use of preserved glaucoma eyedrops even without discontinuing causative eyedrops.

## Figures and Tables

**Table 1:** Patient demographics.

	Total	Group 1	Group 2	Group 3
Number of patients	55	18	22	15
Age (Mean ± SD ^1^, range)	64.1 ± 10.9, 36 to 81	62.6 ± 10.7, 36 to 79	67.5 ± 10.3, 45 to 80	59.7 ± 11.8, 40 to 81
Male (%)	17 (30.9%)	4 (22.2%)	6 (27.3%)	7 (46.7%)
Diagnosis				
POAG ^2^	16 (29.1%)	5 (27.8%)	7 (31.8%)	4 (26.7%)
NTG ^3^	28 (50.9%)	8 (44.4%)	11 (50.0%)	9 (60%)
NVG ^4^	3 (5.5%)	1 (5.6%)	2 (9.1%)	0
Uveitic glaucoma	8 (14.5%)	4 (22.2%)	2 (9.1%)	2 (13.3%)
Medication history				
monotherapy	36 (65.5%)	12 (66.7%)	15 (68.2%)	10 (66.7%)
combination	19 (34.5%)	6 (33.3%)	7 (31.8%)	5 (33.3%)
Medication duration				
<1 year	11 (20.0%)	3 (16.7%)	5 (22.7%)	9 (60.0%)
≥1 year	44 (80.0%)	15 (83.3%)	17 (77.3%)	6 (40.0%)

^1^ SD, standard deviation; ^2^ POAG, primary open angle glaucoma; ^3^ NTG, normal tension glaucoma; ^4^ NVG, neovascular glaucoma.

**Table 2:** Change of ocular surface parameters after treatment in each group.

	Group 1				Group 2				Group 3			
	Mean ± SD ^4^			*p*-Value ^5^	Mean ± SD			*p*-Value ^5^	Mean ± SD			*p*-Value ^5^
	Initial	1 m	2 m		Initial	1 m	2 m		Initial	1 m	2 m	
Schirmer I	4.2 ± 2.4	4.7 ± 3.3	5.7 ± 5.2	0.9815	3.7 ± 1.5	4.5 ± 3.0	5.4 ± 2.7	0.0702	4.7 ± 3.9	5.5 ± 3.0	5.5 ± 2.2	0.2510
TBUT ^1^	3.2 ± 1.6	3.9 ± 1.9	5.6 ± 2.0 *	0.0020	3.4 ± 2.0	3.6 ± 1.7	4.1 ± 1.7 *	0.0195	2.8 ± 2.3	4.2 ± 2.7	3.1 ± 2.1	0.2744
OSS ^2^	5.7 ± 2.5	4.2 ± 2.4	1.9 ± 1.3 *	<0.0001	5.5 ± 2.9	2.9 ± 1.2 *	1.6 ± 1.3 *	<0.0001	5.1 ± 2.3	3.1 ± 1.8	4.5 ± 1.9	0.0480
Meiboscore	1.5 ± 0.9	1.5 ± 0.9	1.5 ± 0.9	1.000	1.5 ± 1.0	1.4 ± 1.0	1.4 ± 1.0	0.9774	1.3 ± 1.2	1. 5 ± 1.1	1.4 ± 1.2	0.8909
Meibum quality	1.2 ± 1.0	1.1 ± 1.0	0.6 ± 0.7	0.1098	1.3 ± 1.2	1.0 ± 0.8	0.8 ± 0.7	0.4023	1.1 ± 1.2	0.8 ± 0.9	1.1 ± 1.2	0.7812
OSDI ^3^	51.9 ± 24.8	43.5 ± 19.6	30.0 ± 16.4 *	0.0037	52.2 ± 22.5	35.3 ± 17.5 *	28.9 ± 12.7 *	0.0004	45.1 ± 16.4	35.8 ± 14.8	41.1 ± 16.6	0.0736

^1^ TBUT, tear breakup time; ^2^ OSS, ocular-surface staining; ^3^ OSDI, ocular-surface disease index; ^4^ SD, standard deviation; ^5^ determined by Kruskal-Wallis test (nonparametric analysis of variance); * significant difference compared to the baseline value, which was determined by Dunn’s post-hoc comparison test.
